# Sympathetic Effect of First Dentition

**Published:** 1872-07

**Authors:** N. E. Hollace

**Affiliations:** Boston, Mass.


AMERICAN JOURNAL

OF

DENTAL SCIENCE.

Vol. VI. THIRD SERIES--JULY, 1872. No. 3.

### ARTICLE I.

### *Sympathetic Effect of First Dentition.*

BY N. E. HOLLACE, M. D., BOSTON, MASS.

It has long been observed that during the process of dentition, children are more liable to disease than at other times, and that this is the most fatal period of life. It is probable, however, that the influence of the process has been much exaggerated. It occupies a large portion of that stage of growth when the susceptibilities of the system are most acute by nature, least blunted by habit, and when morbific causes in general must have the greatest influence. It is at this time of life also that exposure usually begins, and the child becomes liable to those diseases, such as measles, whooping cough, &c., which occur but once. It is, moreover, during dentition that the change of diet usually takes place from the milk of the mother to other and unaccustomed kinds of food, and that the maternal milk itself becomes deteriorated by the institution of new or suspended physiological processes. Independently of dentine, therefore, it would be inferred that this period of life must be unusually fatal; and it is an error to ascribe the result exclusively to that process.

Still there can be no doubt that it exercises an important influence, and that its derangements are among the most fruitful sources of danger to infant life. The pressure of the growing teeth upon the surrounding parts almost necessarily occasions some uneasiness, and frequently produces so much irritation and inflammation, as not only to cause great local suffering to the child, but also to call various functions into sympathetic disorder, and even to set on foot serious organic diseases.

This is easily understood when it is considered that in infancy, not only are the susceptibilities most acute, but the sympathies also peculiarly acute, from the necessity in which the system is placed in the rapid development of all its parts, to guard against irregular results by the quick diffusion of impressions from each part to every other.

The investing membrane of the tooth and the gum over it, against which the body presses in its progress, and which is absorbed in consequence of this pressure, are the parts which chiefly suffer; though it is highly probable that the roots also occasion distress during their development, by incommoding the dental nerves. The irritation thus occasioned is probably often productive of tingling, itching, or other vague uneasiness, difficult from pain and even more difficult to be borne; for the child instinctively seeks relief by biting upon hard substances or otherwise pressing them against his gums; the slight pain thus occasioned being more tolerable than the sensation which it displaces. The irritation sometimes occasions serious constitutional disturbance, especially of a nervous character, for such disturbance has been relieved by lancing the gums, when no obvious inflammation existed. The feeling of relief from pressure is experienced, even when the gum is considerably swollen, hot and painful, but when the surface becomes of a deep red, or assumes that vesicular appearance which sometimes precedes the eruption of the tooth, it is usually exceedingly ender to the touch.

During difficult dentition, the saliva flows more freely, the child becomes fretful, peevish, restless and wakeful at night, frequently putting his fingers into his mouth, and sometimes screaming violently and almost incessantly. In some instances the inflammation extends to neighboring parts affecting the absorbent and salivary glands and the mouth becomes hot and dry.

The sympathetic effects of dentition are generally in proportion to the local affection, which is greatest when many teeth are advancing at the same time. They bear also some relation to the constitution and age. Delicate children usually suffer more than the robust, because the process is more protracted and the danger is greater in premature than in late dentition, because the susceptibilities of the system are greater. From the peculiar mobility of the nervous system of infants this is very apt to suffer, especially the brain. Hence restlessness, wakefulness, occasional spasmodic movements, and even convulsions.

The respiratory organs sometimes participate in the nervous derangement, and there is reason to believe that spasm of the glottis and very obstinate cough may originate in this source. But the irritation is most frequently directed to the alimentary canal. Looseness of the bowels is a very common attendant on teething, and, when not severe, may be considered as a salutary effort to relieve or prevent more dangerous affections, especially of the brain. Sometimes, however, it assumes a dangerous character, being attended with vomiting and great prostration, or becoming protracted and wearing out the strength of the patient.

The skin is also a frequent seat of the sympathetic irritation, and various eruptive affections are apt to appear during dentition. Among these a pustular and ulcerated or scabby disorder behind the ears is not uncommon. The circulation generally participates more or less in the irritation, and fever not unfrequently occurs. Besides all these direct results of diseased dentition, it is apt to aggravate almost all co-existing diseases.

TREATMENT.

The child should in general be exposed to cool fresh air, and the bowels, if at all disposed to costiveness, should be kept open by a laxative diet, or by mild cathartics, such as magnesia or castor oil. If there should be much fever with costiveness, the above will be found useful.

The stomach should never be loaded with food, and the diet should be light and of easy digestion, and proportioned to the degree of existing excitement.

Attendant or congenital diseases must be treated as when they occur under other circumstances, reference however, being always had to the fact, that diarrhœa and cutaneous eruptions often afford a safe outlet to the irritation, and should not be too hastily removed, lest this might fall dangerously upon the brain.

The disorder behind the ears is apt to be unsightly and troublesome, and mothers are often anxious for its cure, but it should rather be encouraged than removed. The local treatment of diseased dentition is highly important.

When a mere irritation exists, without much inflammation or disturbance of the health, it may be sufficient to allow the child to chew upon some hard substance, as a smooth piece of wood, rubber or ivory; but when the gums are swollen and any derangement in the functions occurs, incisions should be made without hesitation down to the teeth, so as to divide both the gum and the investing membrane. Incision over the front should be single, but over the double teeth of a crucial form.

Lancing the gums often affords great and immediate relief. It is useful in some measure by the bleeding which ensues, and is therefore not always without benefit, but its chief advantage is the liberation of the tooth, and the removal of the tension of the gum and membrane.

The objection which has been urged against it by some dentists is that the incision often heals before the tooth appears, and thus leaves a scar more difficult to penetrate than the original gum. So far from affording an increased resistance to the progress of the tooth, the scar partakes of that diminished vitality of all new formed parts, which causes them to yield to the absorbent process more readily than the original structure.

It is asserted that lancing the gums is sometimes useful, even when no swelling appears, by relieving the tension and irritation of the parts immediately round the tooth. Some caution however is requisite, as too early a division of the capsules which secretes the enamel may interfere with the proper formation of that structure.

